# The influence of thoracic gas compression and airflow density dependence on the assessment of pulmonary function at high altitude

**DOI:** 10.14814/phy2.13576

**Published:** 2018-03-29

**Authors:** Troy J. Cross, Courtney Wheatley, Glenn M. Stewart, Kirsten Coffman, Alex Carlson, Jan Stepanek, Norman R. Morris, Bruce D. Johnson

**Affiliations:** ^1^ Division of Cardiovascular Diseases Mayo Clinic Rochester Minnesota; ^2^ Menzies Health Institute Queensland Griffith University Southport Queensland Australia; ^3^ Preventive, Occupational and Aerospace Medicine Mayo Clinic Scottsdale Arizona; ^4^ Allied Health Research Collaborative The Prince Charles Hospital Brisbane Queensland Australia

**Keywords:** Airflow density dependence, forced expiratory flows, high altitude, thoracic gas compression

## Abstract

The purpose of this report was to illustrate how thoracic gas compression (TGC) artifact, and differences in air density, may together conflate the interpretation of changes in the forced expiratory flows (FEFs) at high altitude (>2400 m). Twenty‐four adults (10 women; 44 ± 15 year) with normal baseline pulmonary function (>90% predicted) completed a 12‐day sojourn at Mt. Kilimanjaro. Participants were assessed at Moshi (Day 0, 853 m) and at Barafu Camp (Day 9, 4837 m). Typical maximal expiratory flow‐volume (MEFV) curves were obtained in accordance with ATS/ERS guidelines, and were either: (1) left unadjusted; (2) adjusted for TGC by constructing a “maximal perimeter” MEFV curve; or (3) adjusted for both TGC and differences in air density between altitudes. Forced vital capacity (FVC) was lower at Barafu compared with Moshi camp (5.19 ± 1.29 L vs. 5.40 ± 1.45 L, *P *<* *0.05). Unadjusted data indicated no difference in the mid‐expiratory flows (FEF_25–75%_) between altitudes (∆ + 0.03 ± 0.53 L sec^−1^; ∆ + 1.2 ± 11.9%). Conversely, TGC‐adjusted data revealed that FEF_25–75%_ was significantly improved by sojourning at high altitude (∆ + 0.58 ± 0.78 L sec^−1^; ∆ + 12.9 ± 16.5%, *P *<* *0.05). Finally, when data were adjusted for TGC and air density, FEFs were “less than expected” due to the lower air density at Barafu compared with Moshi camp (∆–0.54 ± 0.68 L sec^−1^; ∆–10.9 ± 13.0%, *P *<* *0.05), indicating a mild obstructive defect had developed on ascent to high altitude. These findings clearly demonstrate the influence that TGC artifact, and differences in air density, bear on flow‐volume data; consequently, it is imperative that future investigators adjust for, or at least acknowledge, these confounding factors when comparing FEFs between altitudes.

## Introduction

The effects of high‐altitude exposure on pulmonary function have been studied extensively over the past century. Early work by Paul Bert in a hypobaric chamber (4500 m; Bert 1878), and by Angelo Mosso at Capanna Regina Margherita (4559 m; Mosso 1897), revealed that vital capacity acutely declines with increasing elevation above sea level. This observation has become so pervasive in the literature that one may consider it a “hallmark” of the acute pulmonary response to sojourning at high altitude (Shields et al. 1968; Dramise et al. 1976; Coates et al. 1979; Jaeger et al. 1979; Stockley and Green 1979; Gautier et al. 1982; Welsh et al. 1993; Saldias et al. 1995; Pollard et al. 1996, 1997; Cogo et al. 1997a,b; Hashimoto et al. 1997; Dillard et al. 1998; Mason et al. 2000, 2003; Deboeck et al. 2005; Fischer et al. 2005; Meysman et al. 2005; Senn et al. 2006; Basu et al. 2007; Fasano et al. 2007) – also see Cremona et al. (2002) and Dehnert et al. (2010). On the contrary, the effect of high‐altitude exposure on the forced expiratory flows (FEFs) is less certain. For example, one may find evidence in the literature that FEFs are augmented (Shields et al. 1968; Mansell et al. 1980; Gautier et al. 1982; Welsh et al. 1993; Saldias et al. 1995; Pollard et al. 1996; Wolf et al. 1996; Cogo et al. 1997b; Cremona et al. 2002; Deboeck et al. 2005; Meysman et al. 2005; Fasano et al. 2007; Dehnert et al. 2010; Lalande et al. 2011; Bouzat et al. 2013), unchanged (Wolf et al. 1996; Pollard et al. 1997; Meysman et al. 2005; Basu et al. 2007; Pellegrino et al. 2010; Lalande et al. 2011) or decreased at high altitude (Stockley and Green 1979; Saldias et al. 1995; Cogo et al. 1997a,b; Hashimoto et al. 1997; Basu et al. 2007) depending on the lung volume considered (i.e., peak vs. mid‐expiratory flows, etc.). It is relevant to note that these studies differed on the bases of altitude exposure, subject demographics and history of high‐altitude pulmonary edema, baseline pulmonary disease status, and location (i.e., hypobaric chamber vs. field‐based expeditions). Given such discrepancies between studies, it is not surprising that published data on FEFs at high‐altitude remain equivocal. We contend, however, that other, more fundamental problems may conflate the interpretation of changes in FEFs at high altitude: namely, thoracic gas compression and the air‐density dependence of maximal expiratory flows.

Thoracic gas compression occurs during forced expirations when intrapleural pressures exceed those required for generating maximal, expiratory airflow (i.e., flow limitation; Ingram and Schilder 1966; Sharafkhaneh et al. 2004). Under such conditions of “choked” flow, increases in expiratory muscle effort serve only to compress alveolar gas in a manner which may be predicted by Boyle's law:

(1) $$\Delta V_{\mathrm{TGC}}\left(\%\right)=\left[\left(P_{b}/\left(P_{b}+\Delta P\right)\right)-1\right]\times 100\%$$

where ∆*V*_TGC_ (%) is the relative decrease in thoracic volume due to gas compression, *P*_b_ is barometric pressure, and ∆*P* is the additional pressure contributed by expiratory muscle effort. If expiratory flow is measured at the mouth, then the integrated expired volume will be larger than thoracic gas volume by a degree equal to ∆*V*_TGC_. Therefore, thoracic gas compression results in the underestimation of maximal, forced expiratory mouth flows at a given expired lung volume (Ingram and Schilder 1966; Sharafkhaneh et al. 2004). This artifact has important implications for the assessment of FEFs at sea level. Not only does thoracic gas compression misconstrue the direct interpretation of the maximal flow‐volume envelope, its presence greatly impairs the assessment of bronchodilator efficacy (Sharafkhaneh et al. 2007) and may also underestimate the degree of flow limitation present during exercise (Guenette et al. 2010). It is of note that Equation (1) states that ∆*V*_TGC_ increases as barometric pressure decreases for a given degree of expiratory effort (∆*P*). Accordingly, the artifact incurred by thoracic gas compression, and the consequences, thereof, can only be worsened by sojourning at high altitude (Jaeger 1972).

One may account for thoracic gas compression artifact if expired volumes and thoracic gas volumes are obtained simultaneously with a body plethysmograph. However, the physical burden of transporting such a device to altitude has detracted from its use in the literature, particular for field‐based expeditions. This point is underscored by the relatively few studies having used body plethysmography to assess changes in pulmonary function at altitude (Gautier et al. 1982; Dehnert et al. 2010; Pellegrino et al. 2010). A simpler method for mitigating the effects of thoracic gas compression is to construct a “maximal perimeter” expiratory flow‐volume curve (Olafsson and Hyatt 1969; Guenette et al. 2010; Cross et al. 2012). In brief, a subject performs a series (6–10) of graded, expiratory vital capacity efforts, and the maximal mouth flows observed at each expired lung volume are taken to construct the composite, “maximal perimeter” curve. This approach obviates the need for body plethysmography, requiring that only expiratory mouth flows are recorded. Yet, while the majority of studies have used mouth flows to measure FEFs at altitude, no study has accounted for thoracic gas compression artifact via the “maximal perimeter” approach outlined above. In turn, the effect of thoracic gas compression on the interpretation of pulmonary function at high altitude remains unclear.

The interpretation of changes in FEFs at high altitude is further complicated by the fact that lower barometric pressures decrease air density which, in turn, raises the maximal wave‐speed flow of an airway segment (Dawson and Elliott 1977; Pedersen and Butler 2011). We, therefore, argue, as have others (Pedersen and Butler 2011), that changes in FEFs at high altitude must be interpreted with respect to those expected on the basis of breathing reduced density air alone. To this end, a simple adjustment can be made to the data obtained at high altitude by multiplying the observed FEFs by the square root of the ratio of air densities between the higher and lower altitudes, respectively (Pedersen and Ingram 1987; Pedersen and Butler 2011). In so doing, one facilitates the comparison between what was *observed* at altitude and what was *expected* simply due to the air‐density dependence of FEFs. However, as with thoracic gas compression, few investigators have addressed the issue of air‐density dependence on FEFs obtained at high altitude (Deboeck et al. 2005; Basu et al. 2007; Fasano et al. 2007; Pellegrino et al. 2010). Therefore, the literature is presently lacking a clear demonstration of how air‐density dependence, and thoracic gas compression, may impact on the interpretation of changes in pulmonary function at high altitude.

The aim of this study was to demonstrate the effects of thoracic gas compression artifact, and differences in air density, on the interpretation of changes in pulmonary function at high altitude. We hypothesized that the magnitude of thoracic gas compression would be greater at Barafu camp (high altitude) compared with Moshi. Furthermore, we expected that the interpretation of the pulmonary function response to high altitude would vary according to whether FEFs were adjusted for air density and/or thoracic gas compression artifact.

## Methods

### Participants and Informed Consent

Twenty‐four healthy adults (44 ± 15 year; 176 ± 9 cm; 75 ± 16 kg; 10 women) participated in the present study. Prior to the study, participants underwent health screening and obtained written approval from their physician. Participants were physically active, nonsmokers, with no history of cardiac disease. Three participants were receiving treatment for hypertension (angiotensin II inhibitor (*n *=* *1); angiotensin converting enzyme inhibitor (*n *=* *1); *β*_1_ blocker (*n *=* *1); *α*_1_ blocker (*n *=* *1)), one participant had history of asthma, and two participants were currently on medication for thyroid disorders (Levothyroxine). All participants provided written informed consent to participate in the study, which had been approved by the Institutional Review Board of the Mayo Clinic.

### Experimental overview

**Figure 1:**
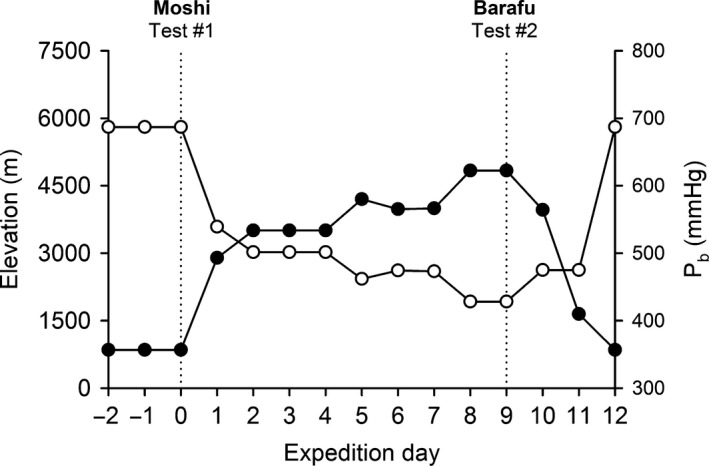
Elevation profile during Mt. Kilimanjaro expedition. The closed circles represent the elevation above sea level, whereas the open circles denote the barometric pressure (*P*_b_) throughout the 12‐day expedition. The vertical dotted lines indicate the days where pulmonary function data were recorded at Moshi and Barafu camp.

Participants were studied during a 12‐day expedition to Barafu camp on Mt. Kilimanjaro, Tanzania (4837 m elevation). An illustration of the elevation profile during this expedition is presented in Figure 1. Participants spent 3 days at Moshi (853 m) before commencing the sojourn to Barafu camp. On the final day at Moshi (Day 0 in Fig. 1), pulmonary function testing was performed on all participants. Participants trekked from Day 0 to Day 8 to arrive at Barafu camp. On Day 9, pulmonary function was obtained once more from all participants. Where possible, testing was performed at the same time of day to minimize diurnal variations in pulmonary function between Days 0 and 9.

### Pulmonary function testing

All pulmonary function tests were performed while participants were seated upright, at rest. The *unadjusted* maximal expiratory flow‐volume (MEFV) envelope was obtained using the routine forced vital capacity (FVC) maneuver. Pulmonary function data were obtained in accordance with current ATS/ERS guidelines (Miller et al. 2005).

### Thoracic gas compression

After standard pulmonary function testing was completed, participants also completed a series (7–9 trials) of vital capacity maneuvers with varying degrees of expiratory effort to mitigate any thoracic gas compression (TGC) artifact – this method has been described in detail elsewhere (Olafsson and Hyatt 1969; Guenette et al. 2010; Cross et al. 2012). Briefly, participants were instructed to inhale to total lung capacity, followed immediately by maximal expiration to residual lung volume. Participants completed this initial expiratory maneuver at 100% of their maximal voluntary effort. Immediately following this effort, participants rapidly inhaled to total lung capacity and, without pause, performed a full exhalation at a slightly lower percentage of maximal expiratory effort. This process was repeated a further 6–8 times at progressively lower percentages of effort (roughly 10% decrements). A “maximal perimeter” MEFV curve was then obtained from the flow‐volume data recorded during these graded vital capacity efforts. This curve represented the participant's MEFV envelope with minimal artifact due to alveolar gas compression.

### Air‐density dependence

The “maximal perimeter” (TGC‐*adjusted*) MEFV curve was further adjusted to determine whether changes in forced expiratory flows between Moshi and Barafu camps could be explained by the decrease in air density associated with sojourning to a higher altitude. We adjusted the MEFV envelope for such air‐density dependence using a similar method to that described by Pedersen and Ingram (1987), wherein maximal expiratory flows obtained under conditions of reduced gas density (e.g., helium–oxygen) are expressed relative to those obtained at another, standard gas density (e.g., normoxia). Importantly, barometric pressure is directly proportional to air density at a constant temperature. Given that our flow‐volume data were standardized to 37°C at each altitude (i.e., BTPS), we took the expedient of using barometric pressure instead of air density in the method outlined by Pedersen and Ingram (1987). Accordingly, the TGC‐adjusted MEFV envelope computed for Barafu was multiplied by a constant factor, given by: *c *= (*P*_Barafu_/*P*_Moshi_)^½^, where *P* is the barometric pressure (mmHg) at either Moshi or Barafu. The value of this constant multiplier was thus: *c *= (428/687)^½^ = 0.789. Because *P* and, therefore, air density at Moshi were the reference point in the above calculation, the value of *c* at this elevation (853 m) was 1.000. Hence, the TGC‐adjusted MEFV envelopes for Moshi data were not multiplied by this factor. It is emphasized that all participants were provided with instructor‐led training on the above respiratory maneuvers, and were afforded several practice attempts on both testing days (Days 0 and 9) before experimental data were collected. It emphasized that the maximal “perimeter” approach does not lend itself easily to adjusting FEV_1_ for TGC artifact. In this study, FEV_1_ was computed using the maximal expiratory flow‐volume envelope (FEV_1,comp_). Because the raw time‐domain flow and volume tracings were not available in the spirometer software suite (see below), we obtained an expiratory time signal by differentiating flow by volume data. The interpolated volume corresponding to 1.00 sec of expiratory time was then reported as FEV_1,comp_ for each level of adjustment.

### Data collection and analysis

Expiratory flow and volume data were collected using a portable handheld turbine spirometer (Spirodoc, MIR©, Roma, Italy) via a USB interface. The device was calibrated across a range of flow rates using a calibrated 3 L syringe (Hans Rudolph Inc., Shawnee, KS) before each test at each altitude. Raw volume/flow data were collected at a sampling frequency of 100 Hz and were expressed in BTPS. The accompanying WinspiroPRO software suite (MIR©, Roma, Italy) was used to acquire and organize data obtained from the graded vital capacity efforts from each subject. Processed flow and volume data for graded vital capacity efforts were exported from the software database into a spreadsheet. These data were exported at a volume resolution of 50 mL due to limitations set by the software suite. To ensure that data density was consistent across individuals of varying vital capacities, flow‐volume data were then resampled to a common base of 100 volume bins per trial. The above‐described interpolation and subsequent TGC and air‐density adjustments were performed using custom‐written software (MATLAB^®^ 2015b, The MathWorks, Inc., Natick).

### Statistical analyses

There were, in total, three levels of adjustments applied to the MEFV curve in this study: (1) unadjusted, (2) TGC‐adjusted, and (3) TGC and air‐density dependence adjusted (TGC+DD). As such, a repeated‐measures analyses of variance (ANOVA) were used to compare differences in pulmonary function variables between the three adjustment levels at a given elevation, separately. Pairwise comparisons were made using the Bonferroni post hoc adjustment. As described above, the TGC+DD adjusted data for Moshi were a duplicate of the TGC‐adjusted data (i.e., *c* = 1.000 at this elevation). Hence, the statistical analyses performed at Moshi simplified to paired *t* tests between unadjusted and TGC‐adjusted datasets. These statistical analyses were designed to primarily address whether TGC was present at each altitude, separately.

To determine whether there occurred more gas compression at higher elevations, it was first necessary to quantify the magnitude of TGC artifact on the MEFV curve. To this end, the absolute (∆) and relative (∆%) differences in the area under the expiratory flow‐volume curve (A_ex_), PEFR, FEF_25%_, FEF_50%_, and FEF_75%_ between unadjusted and TGC‐adjusted levels were computed for each altitude, and were expressed relative to the unadjusted data. Thus, a more negative difference score was interpreted as a greater *underestimation* of forced expiratory flows and, as subsequently, a larger degree of TGC artifact at the corresponding elevation. The Shapiro–Wilk normality test revealed that these difference scores were not normally distributed. Hence, Wilcoxon signed‐rank tests were used to compare difference scores between altitudes (Moshi *v* Barafu) for each variable.

To examine whether high altitude per se affected pulmonary function, change scores (i.e., ∆ and ∆%) between Moshi and Barafu camp were computed for each variable at all adjustment levels (i.e., unadjusted, TGC, and TGC+DD). A repeated‐measures ANOVA was used to evaluate the main effect of adjustment level on changes in pulmonary function observed between the two elevations. Importantly, however, a fourth level consisting of only zeros was added to the ANOVA model. Incorporating this “dummy” level into the model afforded the simultaneous evaluation of whether change scores were different from zero (i.e., did not change between elevations). As above, pairwise comparisons were made using the Bonferroni post hoc adjustment. All statistical analyses were performed using SPSS 20.0 (IBM, Armonk, NY) and were considered significant if *P *<* *0.05.

## Results

The effects of TGC and DD on pulmonary function at Moshi (853 m elevation) and Barafu (4837 m elevation) camps are reported in Table 1. Participants displayed, on average, normal pulmonary function (>80% age‐predicted) at both elevations, irrespective of whether forced expiratory efforts were adjusted for TGC or TGC+DD.

**Table 1:** Impact of thoracic gas compression (TGC) and air‐density dependence (DD) on pulmonary function at Moshi and Barafu camps on Mt. Kilimanjaro

Adjustment	Moshi (853 m)		Barafu (4837 m)		
	Unadjusted	TGC	Unadjusted	TGC	TGC+DD
Dynamic volumes					
FVC (L)	5.40 ± 1.45	–	5.19 ± 1.29	–	–
FVC (% pred.)	114 ± 21	–	110 ± 18	–	–
FEV_1,comp_ (L)	4.07 ± 0.97	4.18 ± 1.01^a^	4.01 ± 0.96	4.25 ± 1.05^a^	3.89 ± 0.97^a^^,^ b
FEV_1,comp_ (% pred.)	109 ± 16	112 ± 16^a^	107 ± 16	113 ± 16^a^	104 ± 15^a^^,^ b
FEV_1,comp_/FVC (%)	76 ± 7	78 ± 7^a^	78 ± 6	82 ± 6^a^	75 ± 7^a^^,^ b
FEV_1,comp_/FVC (% pred.)	96 ± 9	98 ± 9^a^	98 ± 9	103 ± 8^a^	94 ± 9^a^^,^ b
Forced expiratory flows					
PEFR (L sec^−1^)	9.16 ± 2.07	9.30 ± 2.02	9.51 ± 2.26	9.57 ± 2.22	7.55 ± 1.65^a^^,^ b
PEFR (%pred.)	104 ± 16	106 ± 15	108 ± 16	109 ± 16	86 ± 13^a^^,^ b
FEF_25–75%_ (L sec^−1^)	4.53 ± 1.23	4.70 ± 1.25^a^	4.55 ± 1.24	5.28 ± 1.45^a^	4.16 ± 1.14^a^^,^ b
FEF_25–75%_ (% pred.)	132 ± 35	137 ± 35^a^	132 ± 35	153 ± 39^a^	121 ± 31^a^^,^ b
FEF_25%_ (L sec^−1^)	7.35 ± 1.88	7.51 ± 1.97^a^	7.52 ± 1.85	8.07 ± 1.84^a^	6.37 ± 1.45^a^^,^ b
FEF_50%_ (L sec^−1^)	4.55 ± 1.36	4.67 ± 1.32^a^	4.56 ± 1.56	5.32 ± 1.78^a^	4.20 ± 1.40^a^^,^ b
FEF_75%_ (L sec^−1^)	1.77 ± 0.58	2.06 ± 0.71^a^	1.66 ± 0.54	2.22 ± 0.74^a^	1.75 ± 0.59^b^
A_ex_ (L^2^ sec^−1^)	431 ± 99	446 ± 103^a^	439 ± 104	485 ± 117^a^	382 ± 93^a^^,^ b

Values represent means ± SD. TGC+DD, density‐dependence adjustment performed on TGC‐adjusted data; FVC, forced vital capacity; FEV_1,comp_, computed forced expiratory volume in 1 sec; PEFR, peak expiratory flow rate; FEF_25–75%_, mid‐expiratory flows; FEF_25%_, FEF_50%_, and FEF_75%_, forced expiratory flows at 25%, 50% and 75% of FVC; A_ex_, area under maximal expiratory flow‐volume curve. TGC, thoracic gas compression; DD, air‐density dependence

a Significantly different from unadjusted value at the corresponding elevation, *P *<* *0.05.

b Significantly different from TGC‐adjusted value at the corresponding elevation, *P *<* *0.05.

### Thoracic gas compression

**Figure 2:**
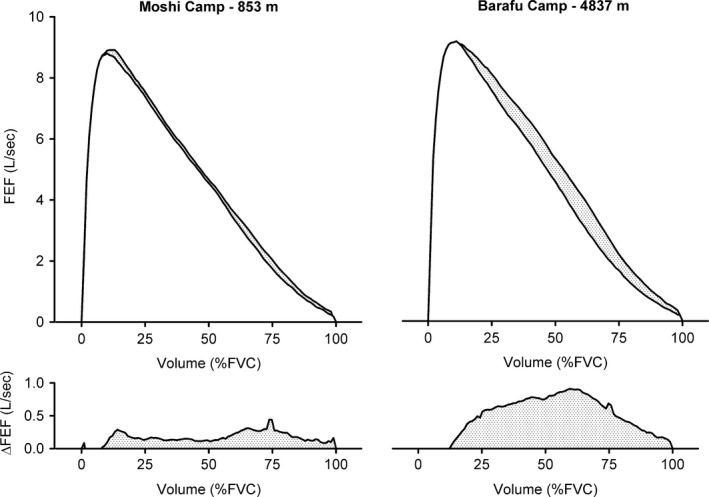
Group‐averaged maximal expiratory flow‐volume curves before and after adjustment for TGC artifact. The lower inner curves in the top panels represent the unadjusted flow‐volume data, whereas the outer curves denote flow‐volume data obtained from the maximal “perimeter” curve (i.e., TGC adjusted data). The stippled area between these two curves indicates the magnitude of TGC artifact. The lower panels illustrate the absolute difference in instantaneous forced expiratory flows between unadjusted and TGC adjusted datasets. Standard deviations are omitted from this figure for clarity. TGC, thoracic gas compression.

**Figure 3:**
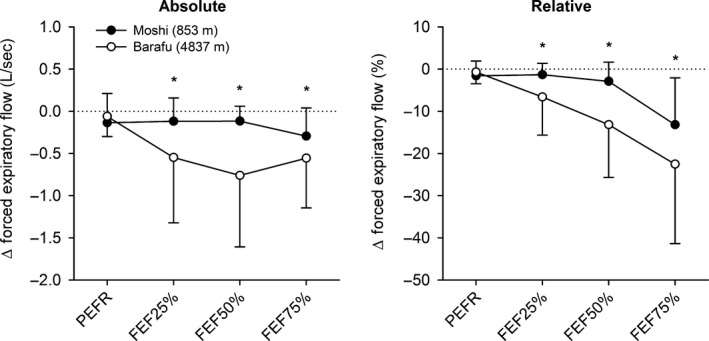
The absolute (left panel) and relative (right panel) magnitudes of TGC artifact at Moshi and Barafu camp on Mt. Kilimanjaro. Data are presented as mean ± SD. PEFR, peak expiratory flow rate; FEF_25%_, FEF_50%_, and FEF_75%_, forced expiratory flows at 25%, 50%, and 75% of forced vital capacity. *Significant difference between elevations (i.e., Moshi vs. Barafu camp) for corresponding forced expiratory flow, *P *<* *0.05. Note that a larger negative value indicates a greater degree of *underestimation* in the corresponding forced expiratory flow due to TGC artifact. TGC, thoracic gas compression.

At both the Moshi and Barafu camps, FEV_1,comp_, FEV_1,comp_/FVC, FEF_25–75%_, FEF_25%_, FEF_50%_, FEF_75%_, and A_ex_ were significantly underestimated due to TGC artifact (*P *<* *0.05). In contrast, however, PEFR did not change despite the TGC correction. The observation that TGC artifact was indeed present at both elevations is further illustrated by the group‐averaged data presented in Figure 2. The degree to which TGC artifact underestimated forced expiratory flows at each elevation is displayed in Figure 3. TGC artifact resulted in a greater magnitude (absolute and relative) of underestimation in FEF_25%_, FEF_50%_ and FEF_75%_ at Barafu compared with Moshi camp (*P *<* *0.05). A_ex_ was also underestimated to a greater extent at the Barafu compared with the Moshi camp (∆–45 ± 49 L^2^ sec^−1^ vs. ∆–16 ± 13 L^2^ sec^−1^, *P *<* *0.05) – a similar finding was observed when this change in A_ex_ was expressed in relative terms (∆–9 ± 8% vs. ∆–4 ± 3%, *P *<* *0.05).

### Air density dependence

At Barafu camp, all pulmonary function variables adjusted for TGC+DD were significantly lower than both TGC‐adjusted and TGC‐unadjusted data (*P *<* *0.05) – excepting that of FEF_75%_ whereby the comparison between TGC‐DD adjusted and unadjusted values did not reach significance (*P *=* *0.67).

### Changes in pulmonary function during ascent to Barafu camp

**Figure 4:**
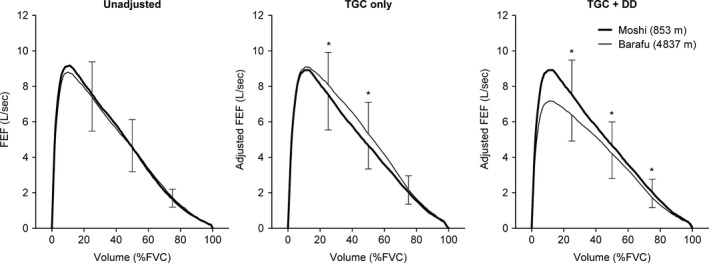
The influence of adjusting for TGC artifact and DD on the interpretation of changes in forced expiratory flows while sojourning at high altitude. Data are presented as mean ± SD for instantaneous forced expiratory flows at 25%, 50%, and 75% of forced vital capacity. TGC + DD: maximal expiratory flow‐volume data adjusted for both TGC and DD; FVC: forced vital capacity. *Significant difference between elevations (i.e., Moshi vs. Barafu camp) for corresponding forced expiratory flow, *P *<* *0.05. The dataset shown in the left panel illustrates the FEFs “as collected” at both elevations (i.e., no adjustments). The dataset in the middle panel reveals that once FEFs are adjusted for TGC artifact, the maximal expiratory flow‐volume curve is, on average, higher at Barafu (high altitude) compared with Moshi (low altitude) – particularly over the first 60% of the forced expiratory volume. The dataset in the right panel (TGC + DD) indicates that while FEFs were *physically* higher at Barafu, they were not as high as expected given the differences in air density between altitudes. Said differently, the TGC + DD dataset at Barafu camp (thickened line) depicts what would be expected if our participants performed their forced expiratory efforts while breathing an inspirate of equal density to that at Moshi. Consequently, the TGC + DD dataset indicates that our participants may have experienced a mild obstructive change in pulmonary function by sojourning at high altitude. TGC, thoracic gas compression; DD, air‐density dependence.

The change in pulmonary function variables between Moshi and Barafu camps for each adjustment level (i.e., unadjusted, TGC, TGC+DD) is reported in Table 2 and are illustrated in Figure 4. For unadjusted data, FVC decreased while PEFR increased upon ascending from Moshi to Barafu camps (*P *<* *0.05) – all other variables did not change between the two elevations. When data were adjusted for TGC, FEV_1,comp_, FEV_1,comp_/FVC, A_ex_, FEF_25‐75%_, FEF_25%_, and FEF_50%_ were together higher at Barafu compared with Moshi camp (*P *<* *0.05). Conversely, by accounting for both TGC and DD (i.e., TGC+DD), all reported variables of pulmonary function significantly “lower than expected” at the higher altitude (*P *<* *0.05). The magnitude of change in FEV_1,comp_, FEV_1,comp_/FVC, A_ex_, FEF_25–75%_, FEF_25%_, FEF_50%_, and FEF_75%_ values between Moshi and Barafu camps was greater for TGC‐adjusted relative to unadjusted data (*P *<* *0.05). Lastly, not only did TGC+DD adjusted data trend in the negative direction with ascent to Barafu camp, this magnitude of change was significantly different from the other two levels of adjustment (i.e., unadjusted and TGC) – excepting that of FEF_75%_ which was different from TGC‐adjusted data only (*P *<* *0.05). There was a trend toward a significant difference between the magnitude of change in FEF_75%_ with altitude between the unadjusted and TGC+DD datasets (*P *=* *0.056).

**Table 2:** Changes in pulmonary function during ascent from Moshi to Barafu camp on Mt. Kilimanjaro

Adjustment	Absolute (∆)			Relative (∆%)		
	Unadjusted	TGC	TGC + DD	Unadjusted	TGC	TGC + DD
Dynamic volumes						
FVC (L)	−0.21 ± 0.33^c^	–	–	−3.2 ± 5.3^c^	–	–
FEV_1,comp_ (L)	−0.06 ± 0.16	0.07 ± 0.33^a^^,^ c	−0.29 ± 0.33^a^^,^ b ^,^ c	−1.3 ± 4.0	1.8 ± 7.0^a^^,^ c	−7.0 ± 6.7^a^^,^ b ^,^ c
FEV_1,comp_/FVC (%)	1 ± 3	4 ± 5^a^^,^ c	−3 ± 5^a^^,^ b ^,^ c	0.2 ± 0.6	0.6 ± 0.8^a^^,^ c	−4.1 ± 6.4^a^^,^ b, c
Forced expiratory flows						
PEFR (L·sec^−1^)	0.35 ± 0.83^c^	0.27 ± 0.93	−1.75 ± 0.85^a^^,^ b ^,^ c	4.1 ± 8.4^c^	3.1 ± 10.0	−18.6 ± 7.9^a^^,^ b ^,^ c
FEF_25‐75%_ (L·sec^−1^)	0.03 ± 0.53	0.58 ± 0.78^a^^,^ c	−0.54 ± 0.68^a^^,^ b ^,^ c	1.2 ± 11.9	12.9 ± 16.5^a^^,^ c	−10.9 ± 13.0^a^^,^ b ^,^ c
FEF_25%_ (L·sec^−1^)	0.17 ± 0.78	0.56 ± 0.92^a^^,^ c	−1.14 ± 0.96^a^, ^b^^,^ c	3.0 ± 10.2	8.9 ± 13.7^c^	−14.1 ± 10.8^a^^,^ b ^,^ c
FEF_50%_ (L·sec^−1^)	0.02 ± 0.77	0.66 ± 1.02^a^^,^ c	−0.46 ± 0.81^a^^,^ b, c	0.6 ± 14.7	14.3 ± 20.9^a^^,^ c	−9.8 ± 16.5^a^^,^ b ^,^ c
FEF_75%_ (L·sec^−1^)	−0.10 ± 0.26	0.16 ± 0.60^a^	−0.31 ± 0.55^b^^,^ c	−5.0 ± 14.4	11.2 ± 31.2^a^	−12.3 ± 24.7^b^^,^ c
A_ex_ (L^2^·sec^−1^)	8 ± 37	38 ± 54^a^^,^ c	−64 ± 47^a^^,^ b ^,^ c	2.1 ± 7.7	8.6 ± 11.6^a^^,^ c	−14.3 ± 9.2^a^^,^ b ^,^ c

Values represent means ± SD.

TGC, thoracic gas compression; TGC + DD, density‐dependence adjustment performed on TGC‐adjusted data; FVC, forced vital capacity; FEV_1,comp_, computed forced expiratory volume in 1 sec; PEFR, peak expiratory flow rate; FEF_25–75%_, mid‐expiratory flows; FEF_25%_, FEF_50%_, and FEF_75%_, forced expiratory flows at 25%, 50%, and 75% of FVC; A_ex_, area under maximal expiratory flow‐volume curve.

a Significantly different from unadjusted value at the corresponding elevation, *P *<* *0.05.

b Significantly different from TGC‐adjusted value at the corresponding elevation, *P *<* *0.05.

c Significantly different from zero, *P *<* *0.05.

## Discussion

The principal aim of this study was to determine the extent to which thoracic gas compression (TGC), and differences in air density, influenced the interpretation of the pulmonary function response to sojourning at high altitude. The novel findings of this study were two‐fold: (1) there was indeed greater TGC artifact on the maximal expiratory flow‐volume curve when forced expiratory efforts were performed at high compared with low elevations; and (2) the interpretation of the effect of high altitude on pulmonary function varied considerably depending on the type of adjustment made to the maximal expiratory flow‐volume envelope (i.e., unadjusted vs. TGC adjusted vs. TGC+DD). These findings together highlight the importance of considering the effects of TGC and air density on the assessment of changes in pulmonary function at high altitude.

### Thoracic gas compression

Because a lower barometric pressure begets a smaller bulk modulus of air (K), we reasoned that air compressibility (i.e., K^−1^) would be greater at higher altitudes. Specifically, we hypothesized that TGC artifact would be larger at Barafu camp relative to Moshi (see Eq. (1)). Our data confirm our original hypothesis, insofar as the underestimation of FEFs due to gas compression was greater at high altitude (Tables 1 and 2; Figs. 2 and 3). Furthermore, TGC led to a larger underestimation of A_ex_ at Barafu camp than Moshi. It is of note that PEFR was seemingly unaffected by our adjustment for TGC artifact at both altitudes. We hypothesize that it is unlikely that ostensibly healthy subjects are capable of generating expiratory muscle pressures of sufficient magnitude and rapidity to limit airflow over the initial portion of the forced expired volume (i.e., <20% FVC; Pedersen and Butler 2011) – the conditions of which are required to produce a nontrivial degree of gas compression artifact.

Although it may appear certain that the greater TGC artifact at high altitude was consequent to an increased gas compressibility, we cannot discount the possibility that high‐altitude exposure, per se, may have facilitated some degree of airways obstruction (discussed later). Such manifestation of airways obstruction may have, in turn, augmented the degree of TGC artifact present during the maximal expiratory maneuvers at high altitude. Thus, with our present data, we cannot partition out the relative importance of these two factors in mediating the larger gas compression artifact at Barafu camp compared with Moshi. Irrespective of this limitation, the following general statement is clear: the artifact incurred by thoracic gas compression is of relatively greater concern when measuring FEFs at higher altitudes.

### Air‐density dependence

The wave‐speed theory of expiratory flow limitation states that the contour of the maximal expiratory flow‐volume curve is determined by the maximal speed at which pressure waves may propagate through the airway tree (for Review see Pedersen and Butler 2011). The quantities affecting the maximal wave‐speed flow (*V*′_*ws*_) of a given airway segment is shown by:

(2) $$V_{\mathrm{ws}}^{\prime }=A^{1/2}{\left(\frac{E}{\rho }\right)}^{1/2}$$

where *A* is the cross‐sectional airway area, *ρ* is gas density, and *E* is the elasticity of the airway segment (i.e., its “tube law”). All else being equal, a reduced barometric pressure proportionally decreases air density, and thereby raises the maximum wave‐speed flow of a given airway segment (Pedersen and Ingram 1987) – a phenomenon commonly referred to as the air‐density dependence (DD) of maximal expiratory flows. Barometric pressure and, by extension, air density at Moshi was roughly 1.61 times greater than that measured at Barafu. The relative increase in maximal expiratory flows due chiefly to a reduced air density at Barafu camp may be estimated by taking the square root of the ratio above (Dawson and Elliott 1977; Deboeck et al. 2005; Basu et al. 2007): that is, 1.61^½^ = 1.27. Consequently, for a constant airway caliber (*A*) and airway wall elasticity (*E*), one may expect FEFs to increase by roughly 30% upon ascent from Moshi to Barafu camp. In this study, we took the opposite approach and assumed that if air density were the only factor affecting maximal flows, then multiplying flow data recorded at Barafu camp by 0.79 (i.e., 1.27^−1^) should result in FEFs equal to those observed at Moshi. Importantly, however, the right panel in Figure 4 reveals that FEFs adjusted for air density (i.e., TGC+DD) at Barafu camp were systematically lower than those observed at Moshi. This observation implies that airway cross‐sectional area (i.e., *A*) and/or the elasticity of the flow‐limiting airway segments (i.e., *E*) were reduced upon ascent to higher altitude in our group of climbers. We caution that the foregoing section should not be taken as evidence that FEFs were *physically* lower at Barafu compared with Moshi. Rather, we infer that, despite there being a general improvement in maximal expiratory flows (i.e., TGC dataset), the augmentation of FEFs was much less than expected given the lower air density at Barafu camp. Stated in other words, if our participants had breathed an inspirate with a density equal to that at Moshi, their FEFs at Barafu camp would have appeared very similar to that illustrated by our TGC+DD dataset in Figure 4.

### Interpreting the changes in pulmonary function at high altitude

We demonstrate here that one may render up to three different interpretations of the pulmonary function response to high altitude depending on whether, and how, pulmonary function data are adjusted (Table 2). It must first be noted that FVC remains unaffected by the method used to adjust pulmonary function data. In that regard, our data are consistent with the wider literature, insofar as FVC declined at the higher elevation (Shields et al. 1968; Dramise et al. 1976; Coates et al. 1979; Jaeger et al. 1979; Stockley and Green 1979; Gautier et al. 1982; Welsh et al. 1993; Saldias et al. 1995; Pollard et al. 1996, 1997; Cogo et al. 1997a,b; Hashimoto et al. 1997; Dillard et al. 1998; Mason et al. 2000, 2003; Deboeck et al. 2005; Fischer et al. 2005; Meysman et al. 2005; Senn et al. 2006; Basu et al. 2007; Fasano et al. 2007). The decline in FVC while sojourning at Barafu camp may have been due to: (1) expiratory muscle weakness limiting the ability to voluntarily reduce lung volume, thereby raising residual volume (Deboeck et al. 2005; Sharma and Brown 2007); (2) hypocapnic‐induced reductions in parenchymal compliance (Cutillo et al. 1974; Pollard et al. 1997); (3) lung fluid accumulation increasing pulmonary elasticity (Jaeger et al. 1979; Welsh et al. 1993); and (4) reduced intraluminal dimensions consequent to increased pulmonary blood volume (Jaeger et al. 1979; Welsh et al. 1993).

When no adjustments were made to the maximal expiratory flow‐volume envelope, the only noticeable finding was that PEFR increased upon ascent to Barafu. If one were to accept this result *prima facie*, it may be stated that high‐altitude exposure did not affect the FEFs occurring over the effort‐*independent* portion of the vital capacity range in our climbers. On the contrary, after the maximal expiratory flow‐volume curve was adjusted for TGC artifact, it was observed that FEV_1,comp_, FEV_1,comp_/FVC, A_ex_, and the mid‐expiratory flows together increased from Moshi to Barafu camp. Finally, when pulmonary function data were adjusted for both TGC artifact and air‐density dependence (i.e., TGC+DD in Table 2), the maximal expiratory flow‐volume envelope at Barafu camp was systematically lower than that recorded at Moshi. Stated in other words, despite accounting for the increased TGC artifact, the rise in FEFs was much less than expected given the ratio of air densities between the two altitudes. Having thus presented three different interpretations of the pulmonary function response to high altitude, the question arises: which of these interpretations is correct?

It is an exceedingly difficult task to assess which of the above adjustments (if any) is most correct based on the wider literature. The matter of drawing reason from the literature is confounded not only by the numerous methods used to collect, adjust, and report pulmonary function data but also due to the varying conditions under which experiments were performed (e.g., field vs. laboratory environments; days/magnitude of altitude exposure; subject demographics). Accordingly, one may appropriate any number of studies to support either of the three interpretations drawn from our data: i.e., FEFs were unchanged at high altitude (Wolf et al. 1996; Pollard et al. 1997; Meysman et al. 2005; Basu et al. 2007; Pellegrino et al. 2010; Lalande et al. 2011), FEFs increased at high altitude (Shields et al. 1968; Mansell et al. 1980; Gautier et al. 1982; Welsh et al. 1993; Saldias et al. 1995; Pollard et al. 1996; Wolf et al. 1996; Cogo et al. 1997b; Cremona et al. 2002; Deboeck et al. 2005; Meysman et al. 2005; Fasano et al. 2007; Dehnert et al. 2010; Lalande et al. 2011; Bouzat et al. 2013), or FEFs were decreased at high altitude (Stockley and Green 1979; Saldias et al. 1995; Cogo et al. 1997a,b; Hashimoto et al. 1997; Fischer et al. 2005; Basu et al. 2007). Rather, we argue that it may be clearer to first decide whether gas compression artifact and/or air‐density dependence is likely to affect the data at hand and, if so, take pains to adjust for their influence. To this end, we have already demonstrated that TGC artifact is greater in magnitude at higher elevations above sea‐level. And it is elementary that air‐density dependence should affect our FEF data given the approximate 60% reduction in air density at Barafu camp relative to Moshi. Hence, we reason that the most appropriate representation of our data is found with the TGC+DD adjusted dataset. Following this line of thought, it may be concluded that our climbers developed a *mixed obstructive–restrictive pattern* in their pulmonary function upon ascent to high altitude (i.e., decreased FVC concomitant with reduced FEFs).

It may be argued that cold‐air exposure, the prevailing airway hypocapnia together conspire to increase airways resistance at high altitude by enhancing bronchomotor tone and hyperresponsiveness (Newhouse et al. 1964; Decramer et al. 1984). However, Gautier et al. (1982) and, more recently, Pellegrino et al. (2010) provided evidence that airway tone and responsiveness may be attenuated (not enhanced) during acute exposure to high altitude in unacclimatazed lowlanders. It is important to note that data in these studies were adjusted for both TGC artifact (via body plethysmography) and differences in air density between altitudes. We, therefore, reason that the mild obstructive pattern observed in our participants was not likely due to an increased bronchomotor tone. Rather, it is more plausible that the mild airways obstruction was consequent to an accumulation of fluid in the lung tissues. Given that no clinical diagnoses of high‐altitude pulmonary edema were reported during the climb, we emphasize that any lung fluid accumulation (if present) was of subclinical magnitude. Nevertheless, lung fluid accumulation may have increased airways resistance (i.e., decreased *A* in Eq. (2)) at high altitude via the encroachment of the airway wall on luminal space and/or a redistribution of wall stresses promoting airway buckling (Pellegrino et al. 2003; Hazel and Heil 2005). Certainly, this hypothesis is supported by previous reports that high‐altitude exposure is associated with: (1) a reduced lung compliance (Jaeger et al. 1979; Pellegrino et al. 2010); (2) raised closing volume and lung mass (Coates et al. 1979; Cremona et al. 2002); (3) an increased transthoracic electrical impedance and the prevalence of ultrasound lung comet tails (Jaeger et al. 1979; Mason et al. 2003; Pratali et al. 2010; Bouzat et al. 2013); and, finally, (4) radiographic evidence of pulmonary congestion (Welsh et al. 1993; Cremona et al. 2002; also see Dehnert et al. 2010). These findings, in conjunction with our observation that FVC declined, are consistent with the rationale that the development of a mixed obstructive–restrictive pattern may have been due to a subclinical (asymptomatic) manifestation of pulmonary congestion at high altitude in our climbers. It is emphasized, however, that the issue of whether subclinical pulmonary edema is a *typical* response to sojourning at high altitude remains debatable (Cogo and Miserocchi 2011a,b; Swenson 2011a,b).

### Practical implications of our findings

It is not often practical to transport a body plethysmograph to high altitude during field‐based expeditions. For that reason, many studies examining the pulmonary function response to altitude exposure have relied solely on mouth flows obtained from pneumotachographs or turbine spirometers. Consequently, data from these studies were not adjusted for TGC artifact. We show here that it is possible to account for TGC artifact by obtaining the “maximal perimeter” expiratory flow‐volume curve (see Methods). This approach imposes only a marginal time burden on participants and investigators, it is relatively simple to implement, and it can be performed with any spirometer so long as the investigator has access to flow‐volume data for postprocessing. Moreover, the adjustment for differences in air density between altitudes requires only knowledge of the corresponding barometric pressures, and a few simple hand calculations. Thus, future investigators seeking to evaluate changes in pulmonary function at high altitude may consider using these approaches, given the relative ease with which these adjustments can be implemented. We further emphasize that adjusting the FEFs for air‐density dependence is only useful once the investigator is assured that TGC artifact has been addressed. If data are not corrected for TGC artifact before air‐density adjustments are performed, a participant's FEFs may be unfairly reduced. For example, in our study, the severity of the obstructive changes in pulmonary function observed at Barafu camp would have been greatly overstated had we only adjusted for air‐density dependence and not TGC artifact (i.e., a DD only dataset).

### Methodological considerations

It cannot be ignored that FVC significantly decreased upon ascending from Moshi to Barafu camp. Consequently, forced expiratory flows at each percentage of FVC (i.e., FEF_25%_, FEF_50%_, and FEF_75%_) must have occurred at different absolute lung volumes. The weight of available evidence suggests that the decline in FVC high altitude is due primarily to an increased residual volume (Dramise et al. 1976; Coates et al. 1979; Jaeger et al. 1979; Mansell et al. 1980; Dillard et al. 1998) rather than a change in total lung capacity (Dramise et al. 1976; Coates et al. 1979; Jaeger et al. 1979). Following this line of thought, the FEF_25%_, FEF_50%_, and FEF_75%_ observed at Barafu camp likely occurred at an absolute lung volume higher than that observed at Moshi (up to 200 mL higher). Despite the potentially higher absolute lung volumes and, by consequence, greater airway conductance (Briscoe and Dubois 1958), FEF_25%_ and FEF_50%_ were “less than expected” at high altitude when data were adjusted for both TGC artifact and air‐density dependence (TGC+DD).

Previous studies have reported that expiratory muscle strength is reduced at high altitude (Deboeck et al. 2005; Sharma and Brown 2007). Considering this evidence, it can be argued that the “less than expected” FEFs (c.f., TGC+DD dataset) observed in our climbers at Barafu camp may have been due to a loss of expiratory muscle strength. Two observations speak against this thesis, however. First, targeted fatigue of the expiratory muscles does not typically cause a reduction in the FEFs over the *effort‐independent* portion of the maximal expiratory flow‐volume curve (Haverkamp et al. 2001). Second, expiratory muscle pressures must have exceeded those required to produce maximal expiratory flow at Barafu camp, seeing that TGC artifact was indeed present at this elevation. We reason that while it is probable that our climbers experienced some degree of expiratory muscle fatigue at high altitude, and that this fatigue may have contributed to the fall in FVC (Deboeck et al. 2005; Verges et al. 2010), it is unlikely to have contributed to the mild obstructive pattern in FEFs observed at Barafu camp. In addition to the above, we may have gained further insight into the mechanisms behind this mild obstructive pattern had we performed bronchoprovocation/bronchodilator testing at each altitude, similar to Dehnert et al. (2010). While these procedures were not feasible in our current study due to the constraints imposed by the expedition itself, future investigators may consider implementing such a strategy, in conjunction with the TGC and air‐density adjustments outlined herein, to provide a clearer interpretation of the changes in pulmonary function observed while sojourning at high altitude.

## Conclusions

The principal finding of this paper was that the interpretation of changes in pulmonary function at high altitude is dependent on whether flow‐volume data are adjusted for gas compression artifact and air‐density dependence. For instance, our unadjusted dataset would suggest no important changes in FEFs had occurred during the climb, whereas the TGC‐adjusted dataset indicated that FEFs were systematically increased by sojourning at high altitude. However, we have argued herein that it is necessary to further adjust data to account for differences in air density between altitudes. In so doing, we report that a mixed obstructive–restrictive pulmonary disorder had manifested in our climbers upon ascent from Moshi to Barafu camp on Mt. Kilimanjaro. In summary, our findings emphasize the need for investigators to be cognizant of the impact that both TGC artifact and air‐density dependence bear on the interpretation of pulmonary function at high altitude.

## Conflict of interest

None declared.
